# Low-glycosylated forms of both FSH and LH play major roles in the natural ovarian stimulation

**DOI:** 10.1080/03009734.2018.1467983

**Published:** 2018-06-12

**Authors:** Leif Wide, Karin Eriksson

**Affiliations:** Department of Medical Sciences, Uppsala University, Clinical Chemistry, University Hospital, Sweden

**Keywords:** FSH glycoforms, LH glycoforms, normal menstrual cycle, ovulation induction, sulfonated N-acetylgalactosamine, sialic acid

## Abstract

**Background:**

The natural ovarian stimulation is mediated by four gonadotrophin glycoforms: FSHtri with three, FSHtetra with four, LHdi with two, and LHtri with three N-glycans. The aim of the study was to determine the serum concentrations of the four glycoforms and their contents of anionic monosaccharides (AMS), i.e. sialic acid (SA) and sulfonated N-acetylgalactosamine (SU) residues throughout the menstrual cycle.

**Methods:**

Serum samples were collected from 78 healthy women with regular menstrual cycles. The serum glycoform molecules were identified by their distributions at electrophoreses. Analyses were also performed after removal of terminal SA. The hormones were measured with time-resolved sandwich fluoroimmunoassays.

**Results:**

The concentration profiles of the four glycoforms were markedly different. FSHtri, which had a 3-fold higher biopotency than FSHtetra, had peak levels on cycle day 5 and at midcycle and nadirs on cycle days 9 and 21–23. FSHtetra had a raised level on cycle days 5–12, followed by a decrease. LHdi and LHtri had similar patterns, but the peak/nadir ratio was much more pronounced for LHdi than for LHtri, 18 versus 4. The numbers of SA residues per molecule were at a maximum around midcycle when the corresponding numbers of SU were at a minimum. The SU/SA ratio was at a minimum on cycle day 12.

**Conclusion:**

The results indicate that the LHdi and the FSHtri molecules play major roles in the natural ovarian stimulation. The SU/SA ratios per molecule favoured a prolonged circulatory half-life of all glycoforms at the midcycle phase. The observations may lead to more successful inductions of ovulation in anovulatory women.

## Introduction

Follicle-stimulating hormone (FSH) and luteinizing hormone (LH) are glycoprotein hormones comprising two dissimilar subunits, termed alpha and beta, which are joined by non-covalent bonding forming two different heterodimers. The protein part of the alpha-subunit is identical for the two hormones, while the protein parts of the beta-subunits differ. N-glycans (oligosaccharides) can be covalently attached to the proteins at asparagine (Asn) residues by an N-glycosidic bond. Fully glycosylated FSH has four glycans (FSHtetra), two on the alpha-subunit and two on the beta-subunit, and fully glycosylated LH has three glycans (LHtri), two on the alpha-subunit, but only one on the beta-subunit.

In a previous report we demonstrated that the two hormones circulate both as fully and as low-glycosylated glycoforms during the menstrual cycle (1). The low-glycosylated FSH was in that report interpreted as being di-glycosylated (FSHdi) based on results from two reports from USA on FSH glycoforms isolated from human pituitaries and urinary preparations (2,3). Later studies by the same research group showed that a majority of the low-glycosylated FSH preparations was tri- and not di-glycosylated (4,5). We have recently shown that the low-glycosylated FSH found in the circulation is tri-glycosylated (FSHtri) and not FSHdi (6). The low-glycosylated LH in the circulation is di-glycosylated (LHdi). The alpha-subunits are decorated with two glycans on all FSH and LH glycoforms (4).

The beta-subunits of the FSHtri molecules may have an N-glycan attached at Asn7 or at Asn24. When FSHtri molecules in human pituitary and urinary extracts were analysed it was concluded that the by far most abundant glycoform of FSHtri possessed an Asn7 N-glycan on the beta-subunit (4,5,7,8).

The glycoforms of FSH and LH are not four single entities. Instead, each glycoform exhibits a considerable heterogeneity due to differences in glycan composition. The isoforms can be separated by electrophoresis due to variation in charge determined by their contents of two terminal anionic monosaccharides (AMS): sialic acid (SA) and sulfonated N-acetylgalactosamine (SU). These terminal AMS residues are decisive for the half-lives of the hormones in human blood circulation (9,10).

This report is a follow-up of our previous study (1) on circulating glycoforms of FSH and LH throughout the menstrual cycle using recently developed methods to identify and characterize these glycoforms (6). The relative frequencies and the concentrations of the two glycoforms of each hormone and their median numbers of AMS, SU, and SA residues per molecule were estimated. With use of the recent data obtained on electrophoretic mobility of the two FSH glycoforms, results by previous biological studies on pituitary FSH could be reinterpreted. This revealed that FSHtri had a 3-fold higher biopotency than FSHtetra. The results indicate that the low-glycosylated gonadotrophin glycoforms play major roles in the natural ovarian stimulation.

## Subjects and methods

### Subjects

As part of the training in clinical chemistry for medical students at Uppsala University Hospital, blood samples were taken from students on an ambulatory basis in the morning during the period 2000 to 2011. All female students who agreed to participate in the research project gave a written consent on analytes accepted to be included and signed a health declaration with information about diseases, medications, alcohol intake, and recent physical activity. Information was given about menstrual bleedings (first day of last menstruation, menstrual cycle length, and regularity over the last year) and hormonal contraceptives. The study was approved by the local Ethics Committee.

One serum sample was used in the study from each of 78 female students, median age 25, range 21–40 y, selected on the criteria that they were apparently healthy, had regular menstrual cycles with a length of 28 ± 2 days, did not use any hormonal contraception, and did not have the common genetic variant of LH (11). They all had serum concentrations of FSH and LH within the reference limits for the day of menstrual cycle. The cycle day was adjusted to a 28-day menstrual cycle. Information about the first day of next cycle was confirmed for samples taken on cycle days 26–28. No serum samples were obtained for cycle days 2 and 4.

### Immunoassay of FSH and LH

The concentrations of FSH and LH in serum samples and in separated fractions after electrophoreses were measured using time-resolved sandwich fluoroimmunoassays (Delfia, PerkinElmer-Wallac Oy, Turku, Finland). The methods permitted measurements of the hormones directly in the 0.075 M veronal (Sigma-Aldrich Chemie Gmbh, Germany) buffer at pH 8.7 eluted from electrophoreses. All sera were initially tested to exclude individuals with the common variant form of LH (11). Gonadotrophin values were expressed in IU/L using the International Standards for pituitary LH (80/552) and FSH (94/632) as reference standards. The detection limits were less than 0.02 IU/L serum, and the interassay coefficient of variation (CV) was less than 3% for both hormones. The detection limit of the two hormones in fractions from electrophoresis was about 100 attogram.

### Biopotency of FSHtri versus FSHtetra

In two studies, reported in the 1980s, human pituitaries were collected 8–48 h post mortem and kept at −20 °C until extracts were made with 0.03 M phosphate buffer (pH 5.6), which were subsequently kept at –20 °C until analysed by electrophoresis (12,13). The pituitary extracts examined were from: (1) a pool of pituitary extracts of 15 men and 15 women aged 18–88 y; (2) a woman aged 56 y; (3) a man aged 38 y; (4) three women aged 16–38 y, mean values; (5) three women aged 55–80 y, mean values; and (6) three men aged 21–42 y, mean values.

The FSH activities in the different fractions after electrophoresis were measured both by *in vitro* bioassay (B) and by radioimmunoassay (I) (12,13). The biological assay method was based upon the estimation of oestradiol produced by cultured Sertoli cells from 10-day-old rats, when incubated with graded doses of FSH in the presence of 19-hydroxyandrostenedione as a substrate (14,15). The relationships between the FSH *in vitro* B/I ratios and the FSH molecular charge were reported in 1986–1987 (12,13). As the electrophoretic technique was the same as that still in use in our laboratory, the electrophoretic positions for FSHtri and FSHtetra could be identified (6). The *in vitro* B/I ratio values were recorded at positions for FSHtri and FSHtetra with negligible overlap in six studies. The positions examined for FSHtri varied from 400 to 460 mAMU (4.5–5.5 AMS/molecule) and for FSHtetra from 570 to 630 mAMU (7.3–8.4 AMS/molecule).

The biopotency of FSHtri was expressed in relation to that of FSHtetra by dividing the *in vitro* B/I ratio of FSHtri with that of FSHtetra. The factors obtained for FSHtri biopotency, versus that of FSHtetra, from the six determinations were 3.0, 2.9, 3.0, 3.2, 2.8, and 3.0, with a mean of 2.98 and a median of 3.0.

### Neuraminidase treatment

The terminal SA residues were removed from the FSH and LH molecules, in serum samples or separated fractions from electrophoresis, by neuraminidase treatment for 24 h at 37 °C, leaving the SU as the only AMS remaining on the molecules, as described (6).

### Frequency of glycoforms of FSH and LH, and AMS residues per glycoform molecule

All serum samples were analysed with an electrophoresis technique using a 0.10% agarose suspension in veronal buffer at pH 8.7. The mobilities were expressed in relation to that of endogenous human serum albumin as Albumin Mobility Units (AMU). The area of eluted gonadotrophin was resolved into peaks at the positions for different numbers of AMS residues per molecule. The electrophoreses and the algorithms used for calculation of the frequencies of the glycoforms of FSH and LH in the serum sample and of the median number of AMS residues per glycoform molecule from the distribution of the hormones were performed as described (6).

### Determination of SU and SA on glycoforms

The number of SU residues and the percentage of SU out of the AMS were determined for each glycoform as described (6). The ratio values of per cent SU out of total AMS per molecule on low- versus fully glycosylated glycoforms were used in the formula to calculate the number of SU and SA per glycoform molecule. The ratio for FSH of %SU on FSHtri versus %SU on FSHtetra at follicular phase and midcycle was 1.351 (range 1.30–1.40; *n* = 7) and at mid-luteal phase 1.26 (range 1.25–1.27; *n* = 3). The corresponding ratio value for LH of %SU on LHdi versus %SU on LHtri at follicular phase and midcycle was 1.257 (range 1.21–1.35; *n* = 7) and at mid-luteal phase 1.045 (range 1.04–1.05; *n* = 3).

### Statistical analyses

The dynamic changes during the ovarian cycle are illustrated as starting four days before the first day of the menstrual cycle, on day 25 in previous cycle, and lasting to day 24 of the menstrual cycle. In order to illustrate the time-related dynamic changes during the cycle, three-day mean values ± SEM, with one day step-wise movements throughout the cycle, were calculated for different data. The calculation started on day 24 in the previous cycle. Data for days 24, 25, and 26 were pooled as the day 25 three-day mean value and for days 25, 26, and 27 as the day 26 mean value, etc.

Data were compared using two-tailed Student’s *t* test. The data passed normality tests. The ratios between number of SA and SU residues per molecule were log-transformed and plotted in the figures as geometric mean ± SEM values. A *P* value less than 0.05 was considered significantly different.

## Results

### FSH and LH serum concentrations

**Figure 1.**
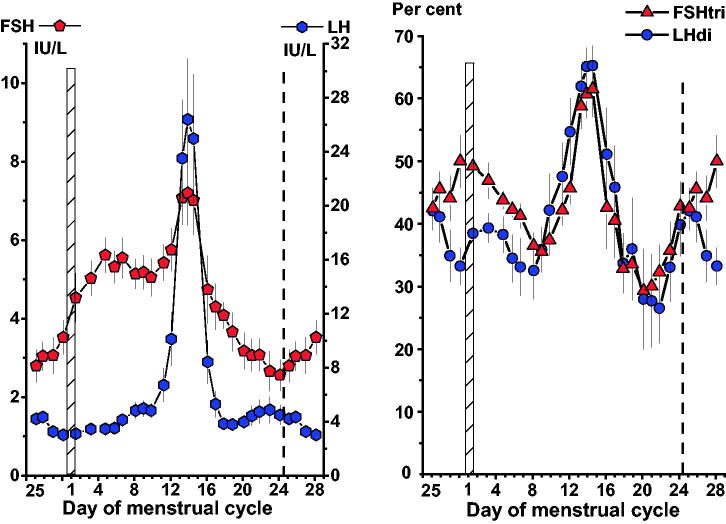
*Left panel:* Concentrations of FSH and LH in serum samples from 78 women with a normal menstrual cycle. *Right panel:* Per cent low-glycosylated forms, FSHtri and LHdi, in these 78 serum samples. Data in this and the following figures are plotted as three-day moving mean values. The day of the menstrual cycle is given and the first day indicated by a vertical hatched bar. The ovarian cycle starts on day 25 of the previous cycle and lasts to day 24 of the menstrual cycle, the end indicated by a vertical dashed line. Mean values ± SEM.

There was a rise of serum FSH during the early follicular phase, from day 25 in the previous cycle to day 5, followed by a slightly decreasing plateau level, shown in Figure 1, left panel. The serum LH concentration increased gradually from day 3 to day 12. The midcycle LH and FSH surges coincided and occurred around day 14. They were followed by rapid decreases of the hormone concentrations. A nadir of FSH was observed on days 23–24.

### Per cent low-glycosylated FSH and LH forms

From the electrophoretic distributions of the gonadotrophins the percentages of low-glycosylated FSH and LH forms, FSHtri and LHdi, respectively, were estimated throughout the menstrual cycle as shown in Figure 1, right panel. The patterns during the menstrual cycle were similar for FSH and LH with pronounced midcycle peaks up to values around 62%–65% low-glycosylated molecules. During the follicular phase there was a gradual decrease of the percentage of FSHtri and LHdi. The lowest values, around 27%–29%, were found during the luteal phase on days 20–22.

### Concentrations of FSHtri and FSHtetra

**Figure 2.**
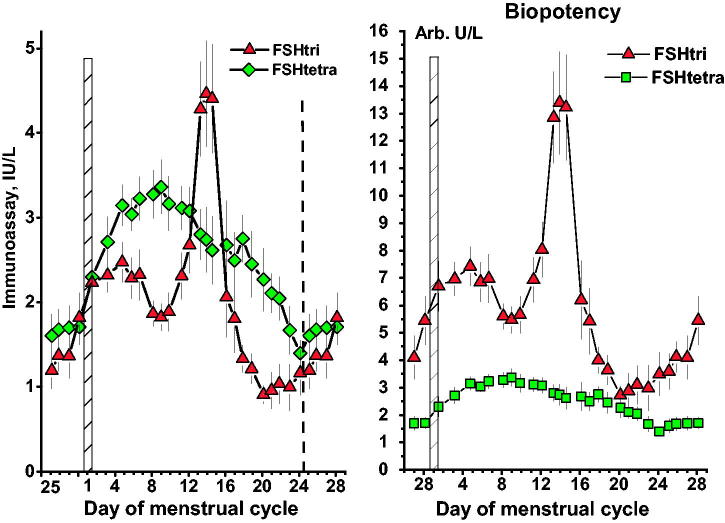
Concentrations of FSHtri and FSHtetra, *left panel*, and their estimated biopotencies, in arbitrary units per L, *right panel*, during the normal menstrual cycle. See also legend to Figure 1.

The concentrations of FSHtri and FSHtetra during the menstrual cycle are shown in Figure 2, left panel. They both increased to a plateau level during mid-follicular phase. After the levels had reached this plateau, the patterns for FSHtri and FSHtetra were completely different. The FSHtri level decreased significantly (*P* < 0.05) from a mean level on days 3–6 of 2.37 IU/L (*n* = 13) to a mean level of 1.78 IU/L (*n* = 9) on days 7–10. This decrease was followed by a pronounced increase to a midcycle peak on days 13–15. After the midcycle peak, the FSHtri level rapidly decreased to the lowest levels on days 20–24, followed by a continuous rise to the mid-follicular phase plateau level. The follicular phase plateau level of FSHtetra lasted until day 12, and after that day continuously decreased until day 24. There was no sign of a midcycle surge of FSHtetra.

### Biopotency of FSHtri and FSHtetra during the menstrual cycle

The expected biopotencies of FSHtri and FSHtetra in serum during the menstrual cycle are shown in Figure 2, right panel. The factor obtained, as described above, for FSHtri biopotency, versus that of FSHtetra, was 3. The values from immunoassay of FSHtri were multiplied by 3 and those of FSHtetra by 1. The results were expressed as arbitrary units per litre (Arb U/L). The three-day mean biopotency values of FSHtri were higher than those of FSHtetra on each day throughout the menstrual cycle.

### Concentrations of LHdi and LHtri

**Figure 3.**
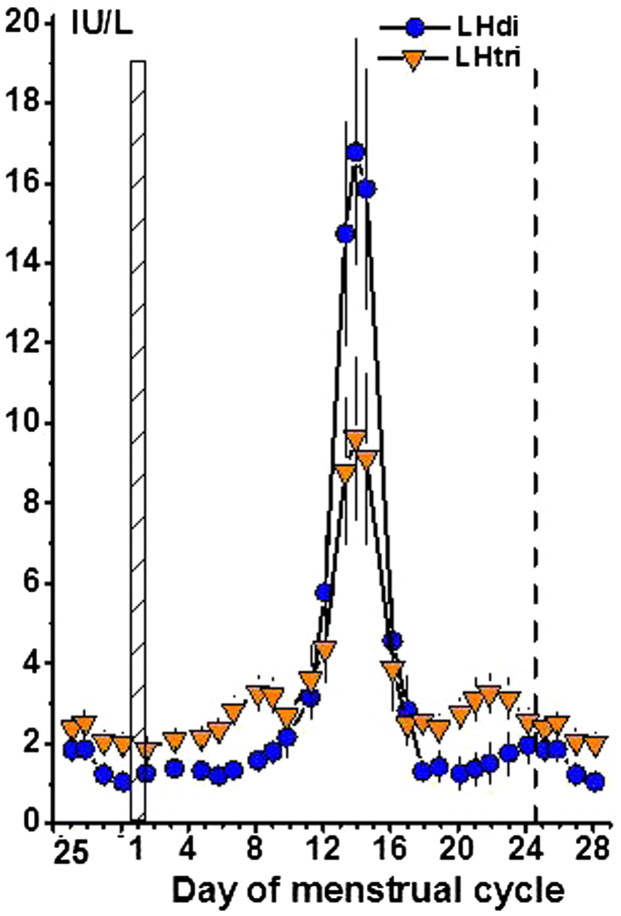
Concentrations of LHdi and LHtri during the normal menstrual cycle. See also legend to Figure 1.

The concentrations of LHdi and LHtri during the menstrual cycle are shown in Figure 3. LHdi and LHtri had similar patterns, but the peak/nadir ratio was much more pronounced for LHdi than for LHtri, a ratio of 18 versus 4.

#### Number of AMS per glycan on the four glycoforms

**Figure 4.**
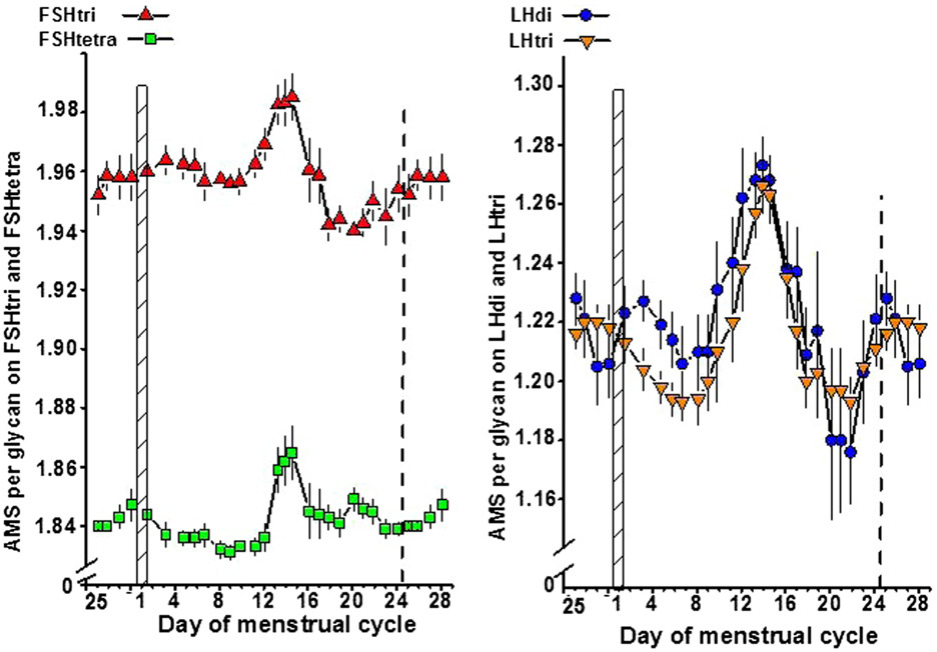
Number of anionic monosaccharide (AMS) residues per glycan on FSHtri and FSHtetra, *left panel*, and on LHdi and LHtri, *right panel*, during the normal menstrual cycle. See also legend to Figure 1.

The number of AMS per glycan on circulating FSHtri, FSHtetra, LHdi, and LHtri during the menstrual cycle is shown in Figure 4. The patterns of the four glycoforms were similar with pronounced increased values at midcycle. Both FSHtri and LHdi had the lowest values during the menstrual cycle at mid-luteal phase. The mean number of AMS on FSHtri was 1.96 and on FSHtetra 1.84, and the difference 0.1175 ± 0.0035 (*n* = 78) was highly significant (*P* < 0.0001) (Figure 4, left panel). The mean number of AMS on LHdi was 1.23 and on LHtri 1.22 (Figure 4, right panel).

#### Number of SU and SA residues per glycoform molecule

**Figure 5.**
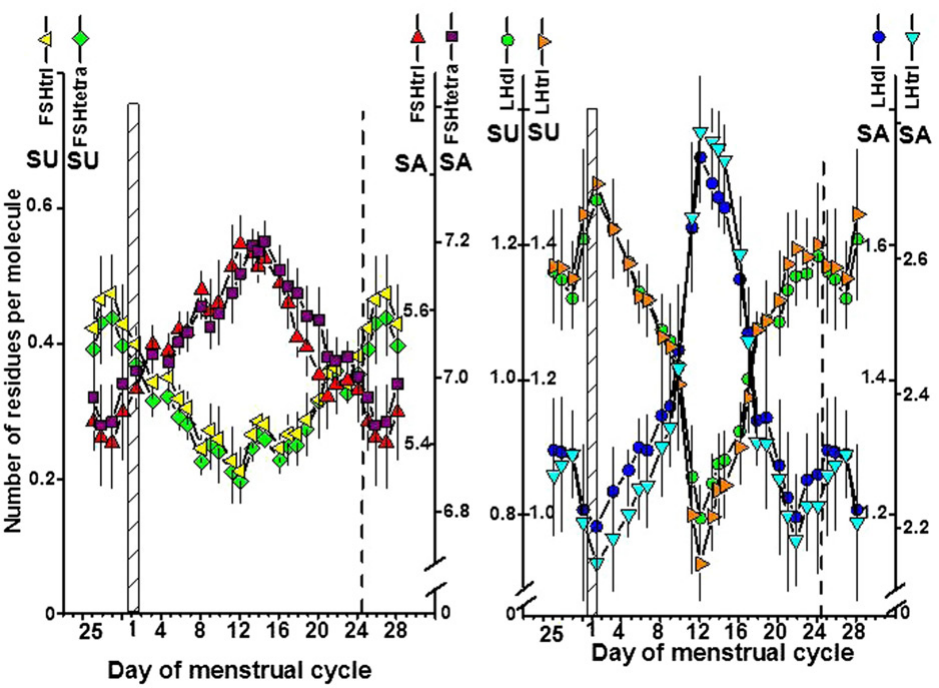
Number of sialic acid (SA) and sulfonated N-acetylgalactosamine (SU) residues per molecule on FSHtri and FSHtetra, *left panel*, and on LHdi and LHtri, *right panel*, during the normal menstrual cycle. See also legend to Figure 1.

The numbers of SU and SA residues per molecule on the four gonadotrophin glycoforms during the menstrual cycle are shown in Figure 5. The intervals used in the seven scales plotted in Figure 5 are identical. For each hormone the patterns of the two glycoforms were similar. The numbers of SU and SA residues per glycoform molecule differed except for SU on FSH which had a similar number of SU residues on the two glycoforms. The number of SU residues decreased to a minimum on day 12, and the number of SA residues increased to a maximum on days 12–15 for both FSH and LH. The changes during the menstrual cycle in residues on LH were more pronounced than those on FSH. The highest level of SU residues on LH was found on the first day of the menstrual cycle, which coincided with a nadir for SA residues.

### Ratios of SU versus SA residues on FSH and LH glycoforms

**Figure 6.**
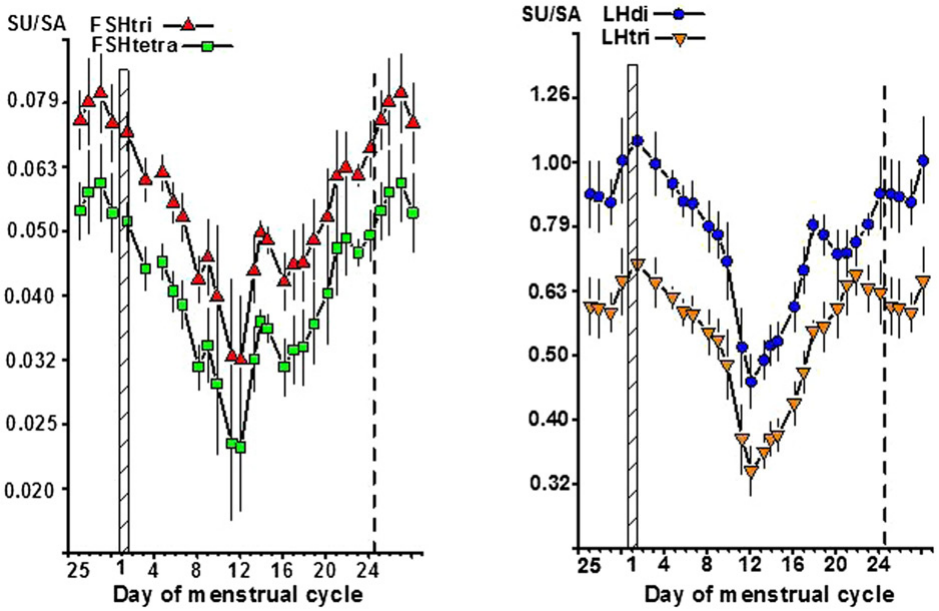
Ratios between number of sialic acid (SA) and sulfonated N-acetylgalactosamine (SU) residues per molecule on FSHtri and FSHtetra, *left panel*, and on LHdi and LHtri, *right panel*, during the normal menstrual cycle. See also legend to Figure 1.

The ratios of SU versus SA residues on the FSH and LH glycoforms during the menstrual cycle are shown in Figure 6, with the SU/SA ratios plotted using a geometric scale. The patterns were similarly V-shaped with a minimum of the SU/SA ratio on cycle day 12. The SU/SA ratios were higher throughout the cycle for the low-glycosylated than for the fully glycosylated forms of both FSH and LH.

### Hormone concentration versus degree of fully glycosylated FSH and LH at midcycle

At midcycle, when the FSH and LH concentrations in serum increased, the frequencies of fully glycosylated FSH and LH decreased. The negative correlation between hormone concentration and per cent fully glycosylated hormones in serum samples, calculated for 34 women on cycle days 9 to 18, was significant for both FSH (Spearman *r* = −0.466; *P* < 0.01) and LH (Spearman *r* = −0.583; *P* < 0.001). As FSH and LH are glycosylated in the same compartment of the pituitary cells, the frequency of FSHtetra was also correlated to the LH concentration, showing a highly significant (Spearman *r* = −0.758; *P* < 0.0001) correlation.

## Discussion

The ovarian cycle is initiated by a rise in FSH which occurs in response to the decline in oestradiol and progesterone in the preceding luteal phase. In the present study the ovarian cycle starts with a rise in FSH on day 25 of the menstrual cycle and lasts to day 24 of the following cycle. A group of follicles in the ovary responds to this rise in FSH and progresses from primordial follicles through the stages of preantral, antral, and preovulatory follicles.

The follicle destined to ovulate is recruited during the first eight days of the menstrual cycle. Oestradiol maintains follicular sensitivity to FSH by aiding FSH in increasing the follicle’s content of FSH receptors. At the mid-follicular phase, there is a gradual fall of FSH levels, which is regarded as a crucial event in the cycle. The dominant follicle survives due to a greater content of FSH receptors. FSH induces LH receptor development on the granulosa cells of the large antral follicles.

A small rise in progesterone prior to ovulation is a signal to the pituitary contributing to the midcycle FSH surge. This FSH surge plays a critical role ensuring ovulation and formation of a normal corpus luteum. A positive feedback of oestradiol on the receptors in the pituitary to gonadotrophin-releasing hormone causes the midcycle surge of LH. After ovulation the levels of FSH and LH rapidly decrease due to a negative feedback by raised levels of progesterone and oestradiol.

In the present study only the low-glycosylated form, FSHtri, exhibits all the crucial changes well known for circulating FSH, while the FSHtetra level slowly declines after a slightly raised level during the follicular phase. The difference between FSHtri and FSHtetra becomes more pronounced when comparing the biopotency levels of the two glycoforms (Figure 2, right panel).

The biopotency of the FSHtri molecules was reported in this study to be three times that of FSHtetra, using an immunoassay to estimate the amounts of the two FSH glycoforms. This finding was based upon the combination of results from three previous publications. The biological activity, measured *in vitro* using Sertoli cells, and the content of FSH, measured with an immunoassay, in fractions of pituitary extracts separated by electrophoresis were reported in 1986–1987 (12,13). Fractions with FSHtri and FSHtetra in those previous studies could be identified as the electrophoretic technique was the same as that used in a recent publication characterizing the glycoforms of FSH (6).

The solid-phase competitive radioimmunoassay used in the studies during the 1980s (16) differed from the non-competitive sandwich fluoroimmunoassay used by us during the following decades. A difference in methodological specificity of the two FSH immunoassays used could have influenced the B/I ratio. However, results with the two FSH assay methods were reported to be almost identical when 10 pituitary extracts were analysed before and after electrophoresis (17).

Our finding of a higher *in vitro* biological activity of FSHtri compared with FSHtetra is supported by some observations reported by Bousfield and co-workers (18,19). They found that low-glycosylated FSH isolated from human pituitary extracts bound more rapidly to rat testis FSH receptors than fully glycosylated FSH (18). Furthermore, the same group found that recombinant low-glycosylated human FSH was more effective than fully glycosylated FSH in stimulating cAMP formation and therewith protein kinase A mediated regulatory phosphorylation in human granulosa cells (19).

The low-glycosylated LH form, LHdi, exhibited much more pronounced changes during the cycle than the fully glycosylated form, LHtri. The nadir-to-peak change in concentration was 18-times for LHdi and only 4-times for LHtri. The glycoform LHdi is less anionic (i.e. less negatively charged) than LHtri (6). The increase of LHdi at midcycle explains our previous observations of less negatively charged forms of LH at midcycle compared with follicular and luteal phase (20). Several studies, reviewed by Bergendah and Veldhuis (21), have shown that the *in vitro* B/I ratio during the menstrual cycle was highest in the late follicular phase and that the less negatively charged LH forms had higher *in vitro* B/I ratios. We therefore postulate that, similar to FSH, the low-glycosylated LH, LHdi, has a much higher biopotency than the fully glycosylated form, LHtri.

**Figure 7.**
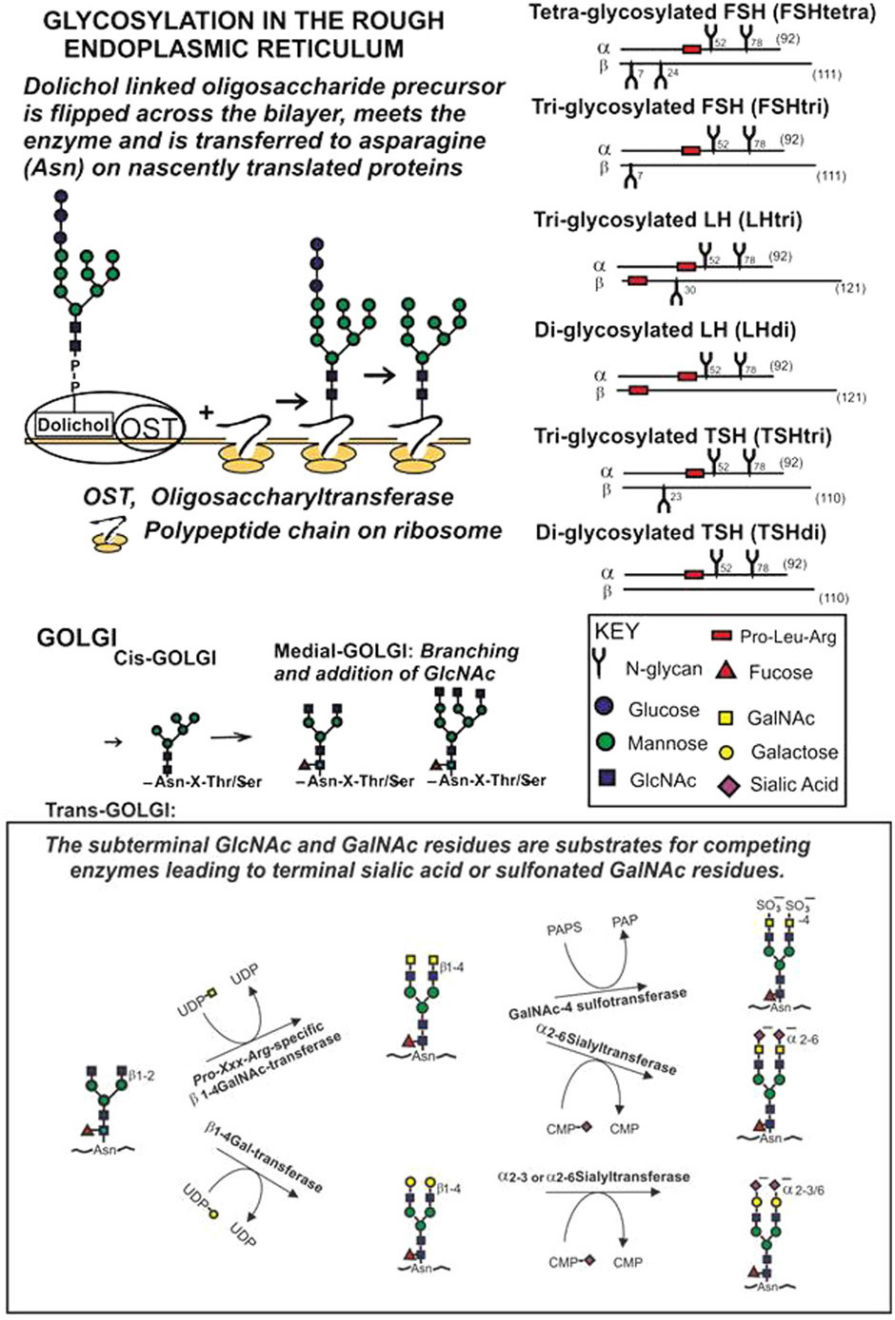
Schematic drawings of the glycosylation in the rough endoplasmic reticulum and the branching followed by the synthesis to terminal sialic acid or sulfonated GalNAc residues in the Golgi of human anterior pituitary cells. The structures of circulating glycoforms of FSH, LH, and TSH are schematically shown. (CMP = cytidine monophosphate; PAPS =3’phosphoadenyl-5’phosphosulphate; UDP = uridine diphosphate).

How is the pituitary production of low- versus fully glycosylated gonadotrophins regulated? The N-glycosylation occurs in the rough endoplasmic reticulum (ER) [see schematic drawing in Figure 7 with nomenclature, pathways, and design from refs. (22–25)]. Dolichol is a special lipid that works as a carrier of the oligosaccharide precursor. The assembly of the dolichol oligosaccharide precursor is formed on the cytoplasmic side of the ER membrane. A flippase then flips the dolichol oligosaccharide precursor across the membrane bilayer to the lumen side of the ER, where enzymes complete the oligosaccharide structure. A protein complex in the ER membrane, termed oligosaccharyltransferase (OST), transfers the oligosaccharide precursor to a gamma amino group of asparagine (-Asn-X-Thr/Ser) on nascently translated proteins.

FSH and LH are glycosylated in the same compartment in the anterior pituitary cells. Both hormones have fully glycosylated alpha-subunits combined with the beta-subunits varying in degrees of glycosylation. The common alpha-subunit is produced in large excess compared with the beta-subunits, ensuring that enough of di-glycosylated alpha-subunits is synthesized. In healthy women of fertile age oestrogens play a major role for a restriction of the glycosylation process of both FSH and LH throughout the menstrual cycles. The availability of the dolichol and the level of the OST activity are rate-limiting factors of the glycosylation process (25), and the oestrogens may act through one, or both, of these two mechanisms. The oestrogen inhibition remains for five to six weeks after a total ovariectomy in young women (unpublished observation).

The finding in this study of a highly significant (*P* < 0.0001) negative correlation between per cent FSHtetra and the LH serum concentration supports the concept that FSH and LH compete during the glycosylation process in the ER. When, at midcycle, the synthesis of the polypeptides of the beta-subunits increases dramatically, without a corresponding increase in glycosylation capacity, the consequence is a large increase of circulating low-glycosylated gonadotrophins. The secretion of these bioactive glycoforms of the gonadotrophins at midcycle is expected to play a fundamental role for the natural ovulatory process.

The number of AMS residues per glycan was larger on FSHtri compared with FSHtetra. A possible explanation is different degrees of branching in the medial-Golgi (Figure 7). The N-glycan at position Asn24 on the beta-subunit of FSHtetra, not present on FSHtri, may have a low degree of branching. An alternative is that the single N-glycan on the beta-subunit of FSHtri, at position Asn7, becomes more branched.

The hormones are secreted in a pulsatile manner, and the compositions of the isoforms continuously change after each pulse. The serum levels depend on both the secretion rates and the disappearance rates of the hormones from the circulation. The terminal SU and SA residues on the glycans are decisive for the half-lives of the hormones in human blood circulation (10,11). The disappearance rate of FSH in the human circulation is mainly regulated by the number of terminal SA residues on the glycans which prolong the survival (10). The disappearance rate of the LH molecules is regulated both by the terminal SA and SU residues on the glycans. Molecules with two or more terminal SU residues are quickly removed from the human blood circulation, suggesting a mannose/sulfonated N-acetylgalactosamine-specific receptor in the human liver similar to that in rodents (26,27).

The mean numbers of SU and SA residues per molecule on each of the four glycoforms changed significantly throughout the menstrual cycle. The numbers of SA residues increased to a maximum around the midcycle period, while the numbers of SU residues were at a minimum around this period of the cycle. The SU/SA ratio was at a minimum on day 12 for each of the four glycoforms. These results indicate that the circulatory half-life of all glycoforms is expected to be short at the beginning and the end of the ovarian and menstrual cycles. The longest half-life in the circulation is expected to be on cycle day 12, followed by a continuous decrease during the next 10–14 days. The moderate increase in serum concentration of FSHtetra during the follicular phase can to a great extent be explained by the increasing number of SA residues on the molecule, resulting in gradually longer circulatory half-life for FSHtetra.

Note that the patterns during the menstrual cycle for the mean numbers of SA residues on the two FSH glycoforms differ considerably from that previously reported for the FSH molecules in serum (1). The explanation of this difference is the large increase in the serum concentration of FSHtri in relation to that of FSHtetra around the midcycle period.

Human gonadotropin preparations have now been used during six decades for the induction of ovulation in anovulatory women. A review of this treatment was presented in the introduction to our previous report (1). These treatments have been highly successful but also associated with a risk for ovarian hyperstimulation and multifetal pregnancies. Mono-ovulation is the aim in the treatment of anovulatory women. It has continuously been a desire to try to mimic the natural ovarian stimulation process more closely to achieve this goal. One prerequisite is then a thorough knowledge about the glycosylation and glycan compositions of serum FSH and LH during the normal menstrual cycle. The gonadotrophins are secreted episodically and both pulse frequency and amplitude do change during the cycle (28). This paper demonstrates for the first time that there are two circulating glycoforms of FSH and two of LH and that the low-glycosylated forms play major roles. All four glycoforms vary in serum concentration and in glycan structures with respect to content of SA and SU throughout the menstrual cycle. With this knowledge, a plausible future treatment alternative, which mimics the natural ovarian stimulation, is to administer mixtures of such recombinant glycoforms of FSH and LH subcutaneously in a pulsatile fashion using a pump.

In conclusion, our results suggest that the low-glycosylated forms of both FSH and LH play major roles in the natural ovarian stimulation. Results from the literature on bioassays of FSH and LH indicate that the low-glycosylated forms of the hormones have a higher biopotency than those which are fully glycosylated. The numbers of SU and SA residues per glycoform molecule change during the cycle with a peak of SA and a nadir of SU at midcycle. The SU/SA ratios per molecule favoured a prolonged circulatory half-life of all glycoforms at the midcycle phase and the shortest half-life at the beginning and end of the cycle. These new observations on the natural ovarian stimulation can lead to better understanding of some pathological conditions, like the polycystic ovarian syndrome and amenorrhea in hypothyroidism. The results may also lead to more successful inductions of ovulation in anovulatory women.
